# Multisite pain and self-reported falls in older people: systematic review and meta-analysis

**DOI:** 10.1186/s13075-019-1847-5

**Published:** 2019-02-22

**Authors:** Victoria K. Welsh, Lorna E. Clarson, Christian D. Mallen, John McBeth

**Affiliations:** 1 Arthritis Research UK Primary Care Centre, Research Institute for Primary Care & Health Sciences, Keele University, Keele, Staffordshire ST5 5BG UK; 2 Arthritis Research UK Centre for Epidemiology, Faculty of Biology, Medicine and Health, The University of Manchester, Manchester, M13 9PT UK

**Keywords:** Musculoskeletal pain, Falls prevention, Public health

## Abstract

**Background:**

Multisite pain and falls are common in older people, and isolated studies have identified multisite pain as a potential falls risk factor. This study aims to synthesise published literature to further explore the relationship between multisite pain and falls and to quantify associated risks.

**Methods:**

Bibliographic databases were searched from inception to December 2017. Studies of community-dwelling adults aged 50 years and older with a multisite pain measurement and a falls outcome were included. Two reviewers screened articles, undertook quality assessment and extracted data. Random-effects meta-analysis was used to pool the effect estimate (odds ratio (OR) and 95% confidence interval (95%CI)). Heterogeneity was assessed by *I*^2^; sensitivity analyses used adjusted risk estimates and exclusively longitudinal studies.

**Results:**

The search identified 49,577 articles, 3145 underwent abstract review, 22 articles were included in the systematic review and 18 were included in the meta-analysis. The unadjusted pooled OR of 1.82 (95%CI 1.55–2.13), demonstrating that those reporting multisite pain are at increased risk of falls, is supported by the adjusted pooled OR of 1.56 (95%CI 1.39–1.74). Multisite pain predicts future falls risk (OR = 1.74 (95%CI 1.57–1.93)). For high-quality studies, those reporting multisite pain have double the odds of a future fall compared to their pain-free counterparts.

**Conclusion:**

Multisite pain is associated with an increased future falls risk in community-dwelling older people. Increasing public awareness of multisite pain as a falls risk factor and advising health and social care professionals to identify older people with multisite pain to signpost accordingly will enable timely falls prevention strategies to be implemented.

**Electronic supplementary material:**

The online version of this article (10.1186/s13075-019-1847-5) contains supplementary material, which is available to authorized users.

## Introduction

Falls are common in older people, and prevalence increases with advancing age, from 20.8% in adults aged 60–69 years to 33.2% for those aged 80 years and older [1]. Known falls risk factors include a history of previous fall, women in the oldest old age groups, particular medication use (for example, benzodiazepines, psychotropics, diuretics and sedatives) and polypharmacy [2]. Specific co-morbidities (including circulatory disease, chronic obstructive pulmonary disease, arthritis) and rising chronic disease burden increase falls risk, along with reduced physical functioning, impaired cognition and visual impairment [2].

Falls are associated with poor outcomes; they are responsible for 65,000 hip fractures annually in the UK [3], cost the National Health Service £2.3 billion per year [4] and can lead to increased need for social support which impacts upon families, communities and employers.

Given the prevalence of falls and their potentially devastating consequences, falls prevention is essential to improving health and well-being of older people. Falls prevention guidelines are based upon management of known risk factors and are widely implemented by health care professionals, yet falls remain a common component of ageing. Multisite pain, defined as pain in more than one part of the body, has been proposed as a novel risk factor for falls [5]. A number of more recent studies have examined this [6, 7]; however, the subject remains under-investigated with comparatively small cohorts and consideration of limited putative confounders of the multisite pain and falls relationship.

As the first study to draw together published evidence, this research tests the hypothesis that multisite pain increases the risk of falls in older people, it seeks to establish the nature of the relationship between multisite pain and falls amongst different community-dwelling populations and to quantify this risk using a systematic review and meta-analysis.

## Methods

The systematic review followed the Preferred Reporting Items for Systematic Reviews and Meta-Analyses (PRISMA) guidelines.

### Search strategy and selection criteria

Seventeen online bibliographic databases were searched from inception until 7 December 2017. The full list of information sources can be found in Additional file 1 and included MEDLINE, Embase, The Cochrane Library, Cumulative Index to Nursing and Allied Health Literature (CINAHL), the British Nursing Index, PsychInfo, Conference Proceedings Citation Index and charity or society websites including AgeUK and the British Geriatric Society. Reference lists of relevant publications were searched, and authors were contacted to obtain further information and to identify additional studies.

Multisite pain was searched using the term ‘pain’ as either an exploded Medical Subject Headings (MeSH) term or as free text; other terms used to capture multisite pain (musculoskeletal diseases, osteoarthritis, arthralgia, pain(ful) hip, pain(ful) knee, pain(ful) ankle, pain(ful) foot) were exploded MeSH terms and searched as free text. All the pain-related terms were combined using the ‘OR’ operator. Fall-related MeSH varied across databases and comprised ‘accidental falls’, ‘falls risk’, ‘falls risk assessment’ and ‘falling’; each of these terms were exploded. The free-text term ‘fall*’ was used to cover fall, falls, falling, fallen and faller. Each of the fall-related search outputs were combined using the ‘OR’ operator. All of the pain-related terms and all of the fall-related terms were then combined using the ‘AND’ operator. The pooled results were limited to those that contained both pain-related and fall-related search terms in either the title, abstract or associated key words.

Searches were limited to human studies only; no other limitations were applied. Studies were excluded if the study population resided in nursing homes or were hospital inpatients or if the pain measure did not quantify the number of pain sites. Authors of studies appearing to have collected information on the number of pain sites or falls but not including this information in the published research were contacted. Studies were excluded when no further information was available.

Articles were included if the population comprised community-dwelling adults aged 50 years and older, a measure of multisite pain was present, the study included a no-pain group and information on falls was available.

Titles and abstracts were screened by two reviewers (VKW, LEC). Methodological quality was assessed by two reviewers independently (VKW, LEC) and a consensus recorded. The Quality in Prognosis Studies (QUIPS) tool, a validated and widely adopted measure used to assess bias in prognostic studies [8], was used to critically appraise the 22 included studies.

This tool was used to assess included studies since key elements of assessing bias in cross-sectional studies are included within this tool and using the same tool for all identified articles enabled comparison between studies.

### Data extraction and analysis

Data extraction from selected studies was undertaken by two reviewers (VKW, LEC) using a study-specific proforma; information was stored in a purpose-designed spreadsheet. Information extracted included country of study setting, type of study, sample size, participant characteristics, recruitment details, inclusion and exclusion criteria, follow-up duration (where relevant), response rate, loss to follow-up, description and classification of multisite pain, potential confounders, falls definition, falls measurement, fall-related outcomes including effect estimates, study conclusion and funding source.

Where possible, data for both unadjusted analysis and the most highly adjusted analysis were extracted from each study. Effect estimates were standardised to odds ratios where possible.

All identified articles were included in the systematic review and examined using narrative synthesis. Random-effects meta-analysis was performed to produce unadjusted and adjusted summary effect estimates (odds ratios (OR) with 95% confidence intervals (CI)). Heterogeneity across studies was measured using *I*^2^ statistic and Cochran’s *Q* test. Publication bias was assessed using funnel plots. For analyses including ten or more studies, Begg’s and Eggar’s tests were used to examine funnel plot symmetry and publication bias. Sensitivity analyses tested the multisite pain-falls relationship in (i) studies that presented adjusted analyses, (ii) longitudinal studies, and (iii) studies considered at low risk of bias. Stata Statistical Software Release 14 was used.

## Results

### Search results

**Fig. 1:**
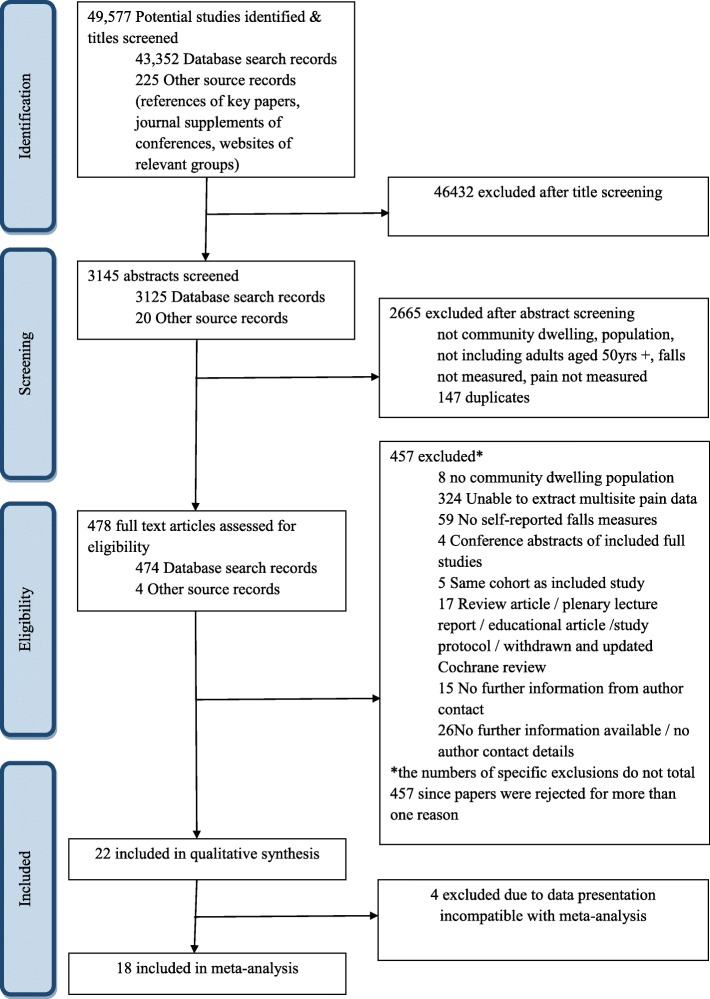
Study flowchart detailing the screening and selection process

The search yielded 49,577 titles, of which 3145 abstracts were screened, 478 full texts were read, 22 studies were included in the systematic review (representing 40,705 participants) and 18 were included in the meta-analysis; the study flowchart is presented in Fig. 1. Two studies included the same study population, one as a cross-sectional study [9] and one a prospective cohort study [10]; both are included in the narrative review.

#### Study characteristics

Additional file 2 presents included studies’ characteristics. A total of 24,935 participants were included from eight countries. Thirty-two percent of studies were from North America, 27% from Japan, 18% from Australia and New Zealand, 14% from the UK and 14% from the rest of the world. A cross-sectional study design was used for 50% of studies, 41% utilised a prospective cohort design and 9% were case-control studies.

#### Pain and falls measures

Pain was defined using different time frames. For example, pain presence ranged from simply ‘the presence of pain’ to ‘pain experienced in the last week’ through ‘pain in the last 12 months’ and ‘pain experienced for at least 3 months in the previous year’. The requirement of current pain alongside a history of pain in the preceding year was used by 14% of studies. Twenty-seven percent of studies used varying definitions to elicit longer-lasting pain rather than acute episodes, for example, ‘persistent pain’, pain on ‘most days’, ‘pain on most days for at least a month’, ‘pain lasting for 1 month or more’, or ‘pain lasting for at least 3 months’.

Severity of pain was included by 9% of studies as part of their classification, and one study used pain associated with radiographic changes to ascertain the pain population.

Multisite pain was defined differently across studies; 41% of studies used body manikins or questionnaires to take account of pain in multiple different body sites’ status, 32% explored pain in specific body parts (for example, upper, middle or lower back and presence or absence of hip pain; or low back pain and hip pain); 27% used presence and number of tender joints as a measure of pain caused by inflammatory conditions and 9% measured number of tender points in fibromyalgia.

The gold standard falls definition provided by the Prevention of Falls Network Europe as ‘an unexpected event in which the participants come to rest on the ground, floor, or lower level’ [11] was used by 32% of studies; 45% did not explicitly state a definition for falls, and the remaining studies provided definitions that were adapted from the gold standard definition.

Fifty-five percent of studies collected retrospective information on falls in the preceding 12 months, 18% collected retrospective information on falls in the preceding 6 months, 23% studies collected prospective information on falls for the 12 months following baseline and 5% collected information on falls for 18 months following the baseline survey. The gold standard method of prospective falls data (use of a falls calendar) was used by 14% of studies.

### Quality assessment

Additional file 3 provides a summary of the risk of bias assessments. Overall, 41% of studies were deemed high risk of bias [12–20], 41% were medium risk [7, 9, 10, 21–26] and 18% were considered to be at a low risk of bias [5, 6, 27, 28]. The most common reasons papers were considered at high risk of bias were unclear description of study participation and study attrition such that bias limitation was unable to be determined. Descriptions of pain measures, falls measures and confounding measures were also limited such that bias potential was deemed partially limited.

### Pain and falls relationship

Additional file 4 presents individual study results, including effect estimates and confounders that were included in calculations. Eighty-two percent of studies demonstrated a statistically significant association between the presence of multisite pain and falls when adjusting for confounding factors [5–7, 9, 10, 12, 13, 15, 16, 18–23, 26, 28]; 18% of studies found no statistically significant relationship between multisite pain and falls after adjusting for confounders [14, 17, 24, 27]. Studies that classified multisite pain by number of pain sites or included a measure of widespreadness found a linear correlation between the number of pain sites and an increasing risk of falls [5, 6, 22, 23, 25].

#### Cross-sectional relationship

Eighty-five percent of studies demonstrated a statistically significant cross-sectional relationship between the presence of multisite pain and previous history of falls when adjusting for confounding factors [6, 7, 9, 12, 13, 15, 16, 18–21]; 15% of studies found no statistically significant cross-sectional relationship after adjusting for confounders [14, 17].

#### Longitudinal relationship

Follow-up periods ranged from 12 months [10, 22, 25–28] through 18 months [5] to 3 years [23, 24]. The retrospective recall periods of falls in the follow-up period ranged from monthly for 12 months [10, 27], four-monthly for 12 months [25, 26], six-monthly for 3 years [23] to the 12 months prior to follow-up [22, 24]. Two studies used daily falls calendars to record falls [5, 28]. All prospective studies reported a trend towards multisite pain increasing the risk of self-reported future fall [5, 10, 22–28]; 22% of those did not reach statistical significance [24, 27].

### Meta-analysis

**Fig. 2:**
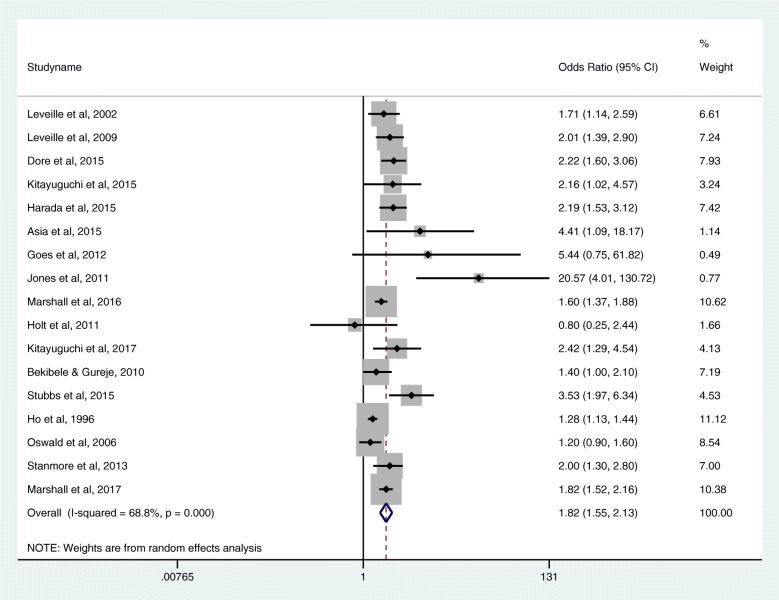
Pooled estimate summary odds ratios for the unadjusted association between multisite pain and falls

As Fig. 2 demonstrates, the pooled estimate summary for the unadjusted association between multisite pain and falls is an OR 1.82 (1.55–2.13). Heterogeneity is likely to have significantly affected the pooled estimate with an *I*^2^ of 68.8% and a Cochran *Q* probability of < 0.01. Additional file 5 demonstrates an asymmetrical funnel plot and Eggar’s test statistics *p* < 0.01 indicating potential publication bias; Eggar’s test *p* = 0.22 indicates potential instability of the test when a small number of studies are included. Those studies presenting only mean tender joint counts or where odds ratios are not able to be calculated are not included in the pooled estimate. Ho et al. [16] had bilateral wrist pain included in the unadjusted analysis, and chronicity multisite measure was used from Kitayuguchi et al. [27].

The relationship between multisite pain and falls persisted in sensitivity analyses. Analysis using risk estimates that have been adjusted to be most representative of real-life clinical scenarios (ten studies, *n* = 14,176) found a summary effect estimate of OR 1.56 (1.39–1.74), *I*^2^ = 0, a symmetrical funnel plot, Begg’s test *p* = 0.09 and Egger’s test *p* = < 0.01.

#### Longitudinal relationship

Analysis including only longitudinal studies (five studies, *n* = 14,633) found an unadjusted summary risk estimate of 1.74 (1.57–1.93), with an *I*^2^ = 0.0%, and a symmetrical funnel plot. Analysis including only prospective cohort studies with adjusted risk estimates to represent real-life scenarios (two studies [23, 28], *n* = 1475) found a summary risk estimate of 1.63 (1.28–2.07), *I*^2^ = 0.0% and a symmetrical funnel plot. Analysis including studies considered at low risk of bias (two studies [5, 28], *n* = 1284) found an unadjusted summary risk estimate of 2.01 (1.54–2.61), *I*^2^ = 0.0% and a symmetrical funnel plot.

## Discussion

### Summary of findings

This systematic review and meta-analysis has found that multisite pain is associated with an increased risk of self-reported falls. Analyses adjusted for confounding factors found that those with multisite pain had increased odds of a self-reported fall of 1.56 (1.39–1.74) compared to those with no pain. When considering only those studies with low risk of bias, the odds of sustaining a future fall for those with multisite pain rose to almost double the odds of those falling with no multisite pain.

### Differences between studies

#### Study designs

Cross-sectional, cohort and case-control studies were included in the review. Whilst prospective cohort studies can establish a temporal relationship, the majority of the studies were cross-sectional, a design that precludes causality. All study designs were included in the meta-analysis to maximise study population and thus provide a more precise summary effect estimate of the multisite pain and falls relationship. Sensitivity analysis using only prospective cohort studies confirmed the relationship between the presence of multisite pain and self-reported falls.

#### Definition of multisite pain

Heterogeneity exists between studies over definitions of multisite pain. Definitions ranged from inclusion of many possible different pain sites using a body manikin to indicate pain sites, to studies specifically enquiring about two sites of pain (for example, low back pain and knee pain). It is therefore possible that respondents reporting no pain in the specified locations are misclassified in the no-pain comparator group when pain might be present, but not in the pre-specified locations. This misclassification may suggest that certain pain phenotypes, for example, knee pain and low back pain, contribute a relatively greater falls risk than other pain phenotypes. This requires further exploration to determine which aspect of multisite pain (for example, the number of pain sites per se, or the pattern of pain experienced) is contributing to future falls risk to enable future targeted research and interventions to reduce falls in older people.

#### Falls outcome measure

The data collection method for self-reported falls ranged from the gold standard of contemporaneously completing daily falls calendars (18% studies) to recall of previous falls (68% studies). Relying on recall of falls may introduce misclassification of fallers as non-fallers and thus lead to an underestimation of falls risk, as demonstrated by Hannan et al. who found that people aged 70 years and older were able to recall only 70% of all falls that had occurred in the previous 3-month period and, of those who fell, 25% were subsequently misclassified as non-fallers [29].

### Strengths and limitations

This systematic review and meta-analysis is comprehensive, drawing on evidence from multiple sources, including research from around the world and including non-English publications.

This study did not explore the association between multisite pain and falls in residential and care home settings. Although the study was designed to exclude such populations, the search strategy did include residential or nursing home residents and no studies were found that explored the relationship between pain and falls within these populations, thus identifying a future research need.

Seventy-eight percent of studies were considered to have a medium or high risk of bias; the most common potential source of bias was due to omitted reporting of response rates and attrition rates with no accompanying explanations for non-response or drop-outs. If the non-response or drop-outs were due to advancing age, poorer health and increased frailty, then the study may underestimate the risk of falls since those more likely to fall have not been included in the study.

The systematic search found only one study exploring the relationship between multisite pain and injurious falls. Welmer et al. found, in a cohort followed over 10 years, that the hazard ratio for pain in two or more sites and future injurious fall (defined by hospitalisation or receipt of outpatient care because of a fall) was 1.79 (1.19–2.69) in their adjusted analysis [30]. The link between multisite pain and subsequent injury or secondary health care requirement has not yet been confirmed in the literature and the association between multisite pain and falls requiring primary health care remains unknown. Further large prospective studies are therefore required to confirm the role of multisite pain as a risk factor for future self-reported falls and to explore the association between multisite pain and falls requiring primary and secondary care utilisation to enable economic analyses to be undertaken.

### Implications for research and clinical practice

This meta-analysis confirms those early study findings that multisite pain is associated with an increased risk of future self-reported falls and there are clear biological pathways to explain this link. For example, pain is associated with mobility limitation which in turn leads to more sedentary behaviour, loss of muscle power and thus an increased risk of falls [2]. Further work must now be undertaken to ascertain the relationship between multisite pain and future falls requiring primary and secondary health care use to determine the level of healthcare use associated with such falls and enable health service planning and organisation to help meet the needs of older people at risk of falls on a population level.

## Conclusions

This systematic review and meta-analysis has found that multisite pain is associated with an increased risk of future self-reported fall. Older people with multisite pain must therefore be considered at increased risk of falls. This is an important public health message to disseminate to older people who can self-identify with multisite pain and seek further guidance from health care professionals to reduce their risk of falls. Health and social care professionals who are regularly reviewing older people are advised to identify those with multisite pain and signpost accordingly so that falls prevention management strategies can be implemented in line with current guidelines.

## Additional files

Additional file 1: Systematic review and meta-analysis: databases and data sources searched. (docx 20 kb)

Additional file 2: Study characteristics for the studies included in the systematic review. (docx 31 kb)

Additional file 3: Summary of the risk of bias assessments using the Quality in Prognostic Studies tool. (docx 23 kb)

Additional file 4: Multisite pain and falls: individual study results. (docx 26 kb)

Additional file 5: Funnel plot of publications examining the unadjusted relationship between multisite pain and falls for cross-sectional, cohort and case-controlled studies. (docx 22 kb)

## Abbreviations

CI: Confidence interval
CINAHL: Cumulative Index to Nursing and Allied Health Literature
MeSH: Medical Subject Headings
OR: Odds ratio
PRISMA: Preferred Reporting Items for Systematic Reviews and Meta-Analyses
QUIPS: Quality in Prognosis Studies
