# Supplementary material for: Multisite pain and self-reported falls in older people: systematic review and meta-analysis
Source: Arthritis Res Ther. 2019 Feb 22;21:67. doi: 10.1186/s13075-019-1847-5 (PMC6387492; doi:10.1186/s13075-019-1847-5)
Supplement: Supplementary file 1 — Systematic review and meta-analysis: databases and data sources searched. (docx 20 kb) [file 13075_2019_1847_MOESM1_ESM.docx]

**Additional File 1: Systematic review and meta-analysis: databases and data sources searched**

| Platform | Database (dates) |
| --- | --- |
| OVID Sp | Medline (1946 to present)  Medline in-process and non-indexed  Embase (1980 to present)  AMED (inception to present)  HMIC Health Management Information Consortium: Department of Health, NICE and King’s Fund (inception to present)  PsychInfo (1806 to present) |
| NHS Healthcare Databases Advanced Search | BNI (1992 to present)  CiNAHL (1981 to present) |
| Web of Science | the Science Citation Index Expanded (SCI-EXPANDED) (from inception to present)  Social Sciences Citation Index (SSCI) (From inception to present)  Conference Proceedings Citation Index-Science (CPCI-S) (From inception to present)  Conference Proceedings Citation Index-Social Science and Humanities (CPCI-SSH) including conference proceedings of the British Geriatrics Society, The American Geriatrics Society, The Gerontological Society of America and the International Association for the Study of Pain (from inception to present) |
| Keele University interface | Ageline (inception to present) |
| Miscellaneous | Cochrane Database of Systematic Reviews (inception to present)  Cochrane DARE database (inception to present)  TRIP database (inception to present)  The Electronic Thesis Online Service (EthOS) (1990 to present) |
| Charity & Society websites | AgeUK  Arthritis Research UK  British Geriatrics Society  The American Geriatric Society  The Gerontological Society of America  International Association for the Study of Pain |
