# Supplementary material for: Multisite pain and self-reported falls in older people: systematic review and meta-analysis
Source: Arthritis Res Ther. 2019 Feb 22;21:67. doi: 10.1186/s13075-019-1847-5 (PMC6387492; doi:10.1186/s13075-019-1847-5)
Supplement: Supplementary file 2 — Study characteristics for the studies included in the systematic review. (docx 31 kb) [file 13075_2019_1847_MOESM2_ESM.docx]

**Additional File 2: Study characteristics for the studies included in the systematic review**

| **Author** | **Study setting** | **Study type** | **Study population** | **Pain measurement and classification** | **Falls measure** | **Funding source** |
| --- | --- | --- | --- | --- | --- | --- |
| **Asai et al, 2015** | Japan, local community association centre | Cross sectional survey | N=112  Men and women with normal motor function  Mean age 73.5 years (68.9 – 78.1 years) | Presence of pain at back, hip, knee, foot or toe, lasting 1 month or more in the previous year and also present in the previous month.  Number of pain sites counted and grouped into no pain, single-site pain and 2 or more pain sites | Retrospective: falls in the preceding 12 months | This study was supported by a Grant-in-Aid for  Young Scientists (B) (22700685) from KAKENHI in Japan. |
| **Bebikele& Gureje, 2010** | Nigeria; community | Cross-sectional | n = 2096  Men and women aged 65 years and older  Mean age of fallers 75.2 years; mean age of non-fallers 75.1 years | Pain in back, neck, chest, joints, headache and ‘persistent pain in any other part of the body’ experienced in previous 12 months.  No pain, single site pain (pain in a single location) and multisite pain (pain in two or more locations) | Retrospective: falls in the previous 12 months | Wellcome Trust |
| **Brenton-Rule et al, 2016** | New Zealand rheumatology outpatient department | Cross sectional study | N= 201  Men and women  Mean age 64.7 years (SD 11)  Rheumatoid arthritis diagnosis according to the 2010 American College of Rheumatology / European League Against Rheumatism classification criteria [1]. | Tender joint count: total and lower limb. | Retrospective: falls in the past 12 months and number of falls | Arthritis New Zealand and the  Health Research Council of New Zealand |
| **Brenton-Rule et al, 2017** | New Zealand rheumatology outpatient department | Prospective cohort | N= 201  Men and women  Aged 18y and older  Mean age 64.7 years (SD 11)  Rheumatoid arthritis diagnosis according to the 2010 American College of Rheumatology / European League Against Rheumatism classification criteria [1]. | Tender joint count | Falls experienced over the 12 month period following baseline study visit. Participant recorded falls on monthly falls calendar and follow up telephone calls. | Arthritis New Zealand and the  Health Research Council of New Zealand |
| **Dore et al, 2015** | United States, community | Prospective cohort study | n = 1619  men and women, African-American or Caucasian  Absence or presence of symptomatic OA as defined by presence of pain, aching, or stiffness on most days, and radiographic evidence (Kellgren-Lawrence grade of 2 or more [2]) of OA in the same joint.    mean age 62 years (range 45-89) | Symptomatic OA for left or right hip or knee - presence of pain, aching or stiffness on most days and associated with radiographic changes. Categorised into symptomatic OA / mild symptoms / moderate or severe symptoms and Kellgren/Lawrence grade >=2 at hip or knee. | Retrospective recall of falls of any type in the previous 12 months and number of falls. | National Center for Advancing Translational Sciences/NIH, CDC/Association of Schools of Public Health and the National Institute of Arthritis and Musculoskeletal and Skin Diseases. |
| **Furuya et al, 2009** | Japan; outpatient rheumatology clinic | Cross-sectional | n = 4996  Men and women with who meet the 1987 classification criteria for rheumatoid arthritis developed by the American College of Rheumatology [3].  Median age 60 years (range 49-74.8 years) | Presence and total number of tender joints | Retrospective: falls in the previous 6 months | ‘36 pharmaceutical companies’ and a grant from the Japanese Osteoporosis Foundation |
| **Goes et al, 2012** | Brazil; inpatient rheumatology ward and local community | Case-control | n = 32 (16 fibromyalgia, 16 health controls matched for BMI, age and physical activity)  Women  Cases: ‘fibromyalgia diagnosed according to the 1990 American College of Rheumatology criteria’ [4].  Healthy controls  Age range 29 – 50 years; mean age fibromyalgia group 41.5years (SD 5.92); mean age control group 40.4 years (SD 6.45) | Presence of pain on the day of study: presence of tender points in lower limbs and general pain | Falls in the previous 6 months | Grant from the Coordination for the Improvement of Higher Education Personnel, Brazil |
| **Harada et al, 2015** | Japan, community setting | Cross sectional survey | n=1351  men and women able to partake in physical activity  aged 65-74 years | Experience of knee pain or low back pain in the past month | Retrospective: falls in the previous 12 months | Grant-in-Aid for Research Fellows of the Japan Society for the Promotion of Science; Waseda University Grant for Special Research Projects; Global COE Program “Sports Sciences for the Promotion of Active Life” from the Japan Ministry of Education, Culture, Sports, Science and Technology. |
| **Hayashi-bara et al, 2010** | Japan; outpatient rheumatology clinic | Prospective cohort | n = 84  Women with ‘definitive diagnosis’ of rheumatoid arthritis  Mean age 65.3 (range 50-82 years) | Tender joint count | Prospective: monthly falls calendars completed daily for 12 months | Grant-in-Aid from the Ministry of Education, Culture, Sports, Science and Technology of Japan |
| **Ho et al, 1996** | Hong Kong; nationwide | Cross-sectional | n = 1947  Men and women  Aged 70 years and older | Musculoskeletal pain in different sites experienced in the previous month  Single site and multisite pain measurements | Retrospective: falls in the previous 12 months | The Croucher Foundation, United Kingdom |
| **Holt et al, 2011** | New Zealand and Australia; community chiropractor clinics | Cross-sectional | n = 101  Men and women  Mean age 72 years (standard deviation 5.9, range 65-92 years) | Presence or absence of back and/or neck pain  Multisite pain (pain in the neck and back); single site pain (pain in back or neck); no pain | Retrospective: falls in the previous 12 months | Grant from the Australian Spinal Research Foundation |
| **Jones et al, 2011** | United States;  Outpatient fibromyalgia clinic & local university | Case-control | n = 52 (27 fibromyalgia, 25 health controls)  Men and women  Cases: ‘fibromyalgia diagnosed according to the 1990 American College of Rheumatology criteria’ [4].  Healthy controls  Mean age 48.6 years (standard deviation 9.7, range 30-59 years) | Number of painful body regions recorded on body diagram  Mean number of painful body regions in fibromyalgia and control group reported | Retrospective: falls in the previous 6 months | National Institutes of Health, Fibromyalgia Information Foundation |
| **Kitayug-uchi et al, 2015** | Japan, general community attending falls prevention clinic | Cross sectional survey | N = 491  Men and women aged 60 years and older  Mean age 72.2 (66.2-78 years) | How much low back pain and knee pain experienced in the last week: none, mild, severe, very severe. Mild to very severe classed as 'pain' group. | Falls in the last 12 months | Not explicitly stated. |
| **Kitayug-uchi et al, 2017** | Japan, community based | Prospective cohort study | N = 1890  Men and women  Mean age 68.3 years (SD 5.9 years) | Low back pain (LBP) and knee pain (KP) presence. Pain classified into current pain lasting longer than 3 months, current pain lasting less than 3 months, no pain. Multisite pain defined as no chronic LBP or KP, either chronic LBP or KP, both LBP and KP. | Retrospective recall of falls over the past 12 months | This study was supported  by a Grant-in-Aid from the Ministry of Health, Labor and  Welfare of Japan (H20-Junkankitou-Ippan-001). |
| **Leveille et al, 2002** | United States; community | Prospective cohort | n = 940  Older women with disability  Widespread pain group mean age 76.5 years (SD 7.3)  Comparator group mean age 80.2 years (SD 8.1) | Pain in the hand, wrist, back, chest (excluding angina), hip, knee or foot on most days for at least a month in the previous year  Single site pain (moderate or severe lower extremity pain (hip, knee or foot))  Multisite pain (widespread musculoskeletal pain (pain in upper extremities (hand or wrist) and lower extremities (hip, knee or foot) and axial skeleton (back or chest) with at least moderate pain (score >4 on numerical rating scale) in one site)  No pain or only mild pain (score <4 on numerical rating scale) | Retrospective: falls in the previous 6 months prior to follow up; six-monthly follow-up over 3 years | National Institute on Ageing |
| **Leveille et al, 2009** | United States; community | Prospective cohort | n = 749  Men and women  Mean age 78 years (SD 5years) | Pain for at least 3 months in the previous year in hands, wrists, shoulders, back, hips, knees, feet, chest (excluding angina).  Multisite pain (pain in two or more locations); single site pain (pain in a single location); no pain | Prospective: monthly falls postcards completed daily for 18 months | National Institute on Aging  Coding of medication data supported by grant from Pfizer (non of which supported salary, stipends or other funding except salaries of researchers) |
| **Marshall et al, 2016** | USA, community setting | Prospective cohort study | N= 6,841  Women aged 65 years and older  Mean age 73.1 years (standard deviation 5.0, 4.8)for the no pain group and mild pain group. 73.3 years (4.9) for the moderate pain group and 73.7 years (standard deviation 5.1) for the severe pain group | ‘Any back pain in the last 12 months?'. Those reporting 'yes' marked on a drawing where their back pain usually occurred (upper, middle, lower). This was classified into lower back only, upper back only, mid back only, and number of pain sites as 1, 2 and 3. Presence or absence of hip pain | Retrospective recall of fall and number of fall every 4 months following baseline. 12 month follow up | The Study of Osteoporotic Fractures is supported by the National Institute on  Aging through grant numbers R01 AG005407, R01 AR35583, R01 AR35584,  R01 AG005394, R01 AG027574, and R01 AG027576. |
| **Marshall et al, 2017** | USA, community setting | Prospective cohort | N = 5568, 65 years and older  Male | Baseline back pain in the past 12 months in upper, mid or lower back or buttocks. Severity and frequency of back pain. hip or knee pain in the past 12 months | Falls every 4 months after baseline using questionnaire to record new falls and number of falls. 12 month follow up. | National Institutes of Health, the National Institute on Aging, the National Institute of Arthritis and Musculoskeletal and Skin Diseases, the National Center for Advancing Translational Sciences and the NIH Roadmap for Medical Research |
| **Oswald et al, 2006** | United Kingdom; outpatient rheumatology clinic | Cross-sectional | n = 316  Women either satisfying the 1987 American Rheumatology Association criteria for rheumatoid arthritis [3] or had been given a consultant diagnosis of rheumatoid arthritis, undifferentiated inflammatory polyarthritis, or post viral arthritis to explain their symptoms.  Mean age 59 years (SD 13.2) | Presence and total number of tender joints | Retrospective: falls in the previous 12 months | Not explicitly stated |
| **Patel et al, 2014** | United States, community or residential setting | Cross sectional survey | n=7601  men and women  older people receiving Medicare  aged 65yrs and over | Bothered by pain in the last month and indication of pain site on a card | Retrospective: falls in the previous 12 months and how many | National Insitute on Aging through cooperative agreement with the John Hopkins Bloomberg School of Public Health |
| **Stanmore et al, 2013** | United Kingdom, Rheumatology out patient clinics | Prospective cohort study | n=535  men and women  All participants have rheumatoid arthritis defined by 2010 American College of Rheumatology/ European League Against Rheumatism classification for rheumatoid arthritis [1].  mean age 62 years (48-75 years) | Number of swollen or tender joints | Prospective: monthly falls calendars completed daily over 12 months | Arthritis Research UK, Wellcome Trust Clinical Research Facility, Manchester. |
| **Stubbs et al, 2015** | UK, community setting | Cross sectional survey | N = 295  Men and women  Mean age 77.5 years (69.4 – 85.6 years) | Report of musculoskeletal pain present over the past month and for at least 3 months of the previous year. Classified into no chronic musculoskeletal pain (CMP), single site CMP and multisite CMP | Retrospective recall of falls over the preceding 12 months | Vice Chancellor’s scholarship at the University of Greenwich |
| SD = standard deviation; OA = osteoarthritis ACR=American College of Rheumatology  References: [1] Aletaha D, Neogi T, Silman AJ, Funovits J, Felson DT, Bingham CO, et al. 2010 rheumatoid arthritis classification criteria: an American College of Rheumatology/European League Against Rheumatism collaborative initiative. Arthritis Rheum 2010;62:2569–81. [2] The epidemiology of chronic rheumatism. Vol II. Atlas of standard radiographs of arthritis. Oxford: Blackwell Scientific Publications, 1963 [3] Arnett FC, Edworthy SM, Bloch DA, McShane DJ, Fries JF, Cooper NS, et al. The American Rheumatism Association 1987 revised criteria for the classification of rheumatoid arthritis. Arthritis Rheum 1988;31:315–324 [4]. Wolfe F, Smythe HA, Yunus MB, Bennett RM, Bombardier C, Goldenberg DL, et al. The American College of Rheumatology 1990 criteria for the classification of fibromyalgia: report of the Multicenter Criteria Committee. Arthritis Rheumatol 1990;33:160–72. | | | | | | |
