# Supplementary material for: Multisite pain and self-reported falls in older people: systematic review and meta-analysis
Source: Arthritis Res Ther. 2019 Feb 22;21:67. doi: 10.1186/s13075-019-1847-5 (PMC6387492; doi:10.1186/s13075-019-1847-5)
Supplement: Supplementary file 3 — Summary of the risk of bias assessments using the Quality in Prognostic Studies tool. (docx 23 kb) [file 13075_2019_1847_MOESM3_ESM.docx]

**Additional File 3: Summary of the risk of bias assessments using the Quality in Prognostic Studies tool**

| **QUIPS assessment domain** | | | | | | | |
| --- | --- | --- | --- | --- | --- | --- | --- |
| **Author** | **Study participation** | **Study attrition** | **Measure of Pain** | **Measure of Falls** | **Study confounding** | **Statistical analysis & reporting** | **Summary of bias** |
| **Asai et al, 2015** | Unclear | Unclear | Partial | Partial | Partial | Yes | High |
| **Bekibele & Gureje 2010** | Yes | Unclear | Partial | Partial | Yes | Partial | Medium |
| **Brenton-Rule et al, 2016** | Unclear | Unclear | Yes | Partial | Partial | Partial | Medium |
| **Brenton-Rule et al, 2017** | Unclear | Yes | Yes | Yes | Partial | Partial | Medium |
| **Dore et al, 2015** | Partial | Unclear | Partial | Partial | Partial | Yes | Medium |
| **Furuya et al, 2009** | Unclear | Unclear | Partial | Partial | Partial | Partial | High |
| **Goes et al, 2012** | unclear | Unclear | Partial | Partial | Partial | Yes | High |
| **Harada et al, 2015** | Unclear | Unclear | Partial | Partial | No | Yes | High |
| **Hayashibara et al, 2010** | Unclear | Unclear | Yes | Yes | Partial | Yes | Low |
| **Ho et al, 1996** | Unclear | Unclear | No | Partial | Partial | Partial | High |
| **Holt et al, 2011** | Unclear | Unclear | Partial | Partial | No | Partial | High |
| **Jones et al, 2011** | Partial | Unclear | Yes | Partial | No | Partial | High |
| **Kitayugachi et al, 2015** | Unclear | Unclear | Yes | Partial | Partial | Partial | High |
| **Kitayuguchi et al, 2017** | Unclear | Unclear | Yes | Partial | Partial | Partial | Medium |
| **Leveille et al, 2002** | Yes | Unclear | Yes | Partial | Partial | Yes | Medium |
| **Leveille et al, 2009** | Yes | Unclear | Yes | Yes | Yes | Yes | Low |
| **Marshall et al, 2016** | Unclear | Unclear | Yes | Partial | Partial | Yes | Medium |
| **Marshall et al, 2017** | Partial | Partial | Yes | Partial | Partial | Yes | Medium |
| **Oswald et al, 2006** | Unclear | Partial | Yes | Partial | Partial | Yes | High |
| **Patel et al, 2014** | Yes | Partial | Partial | Partial | Partial | Yes | Low |
| **Stanmore et al, 2013** | Partial | No | Yes | Yes | Partial | Yes | Low |
| **Stubbs et al, 2015** | Partial | Unclear | Partial | Partial | Partial | Partial | Medium |
| For each QUIPS domain, studies are assessed for potential sources of bias and scored according to sufficiency to limit potential bias; yes = bias potential is sufficiently limited; partial = bias potential is partially limited; unclear = unable to determine bias limitation; no = bias potential is not sufficiently limited.  Summary of bias risk is scored as high = high risk of bias; medium = medium risk of bias; low = low risk of bias. | | | | | | | |
