# Supplementary material for: Multisite pain and self-reported falls in older people: systematic review and meta-analysis
Source: Arthritis Res Ther. 2019 Feb 22;21:67. doi: 10.1186/s13075-019-1847-5 (PMC6387492; doi:10.1186/s13075-019-1847-5)
Supplement: Supplementary file 4 — Multisite pain and falls: individual study results. (docx 26 kb) [file 13075_2019_1847_MOESM4_ESM.docx]

**Additional File 4: multisite pain and falls: individual study results**

| **Author** | **Multisite pain & risk of falls (OR with 95% CI) compared with a no pain group unless otherwise specified** | **Variables adjusted for in analysis** |
| --- | --- | --- |
| Asia 2015 | Unadjusted: 5.02(1.50-17.88)  Adjusted: 5.31 1.40-21.50) | physical function, fear of falls |
| Bekibele 2010 | 1.2(1.0-1.4) | age and sex |
| Brenton-Rule 2016 | Tender joint count lower limb (mean,SD):  Non-fallers 4.98(6.9)  Fallers 6.63(7.37)  P = 0.04 | unadjusted |
| Brenton-Rule 2017 | Tender joint count  (mean, SD):  Non-fallers 9 (SD 11)  Fallers 14 (SD 14)  P=0.005 | Unadjusted |
| Dore 2015 | Unadjusted:  2.22 (1.60-3.06)  Adjusted:  1 symptomatic joint:  1.53(1.10-2.14)  2 symptomatic joints:  1.74(1.19-2.53)  3-4 symptomatic joints:  1.85(0.96-3.55) | age, BMI, gender, ethnicity, prior falls |
| Furuya 2009 | Per tender joint count increase: 1.39(1.14-1.70) | age, sex, BMI, disease duration, J-HAQ score, ESR, CRP, DAS-28. VAS pain, VAS general health, swollen joint count, total knee replacement or total hip replacement, NSAID use, prednisolone dose, methotrexate use, any osteoporosis drug use, active vitamin D3 use, bisphosphonate use |
| Goes 2012 | 5.44(0.91–32.31) | Unadjusted |
| Harada 2015 | Risk of falls with two pain sites:  Unadjusted: 2.19 (1.53-3.12)  Model 1: 2.19 (1.53-3.12)  Model 2: 1.93 (1.35-2.78) | Model 1: age and sex  Model 2: age, sex, lower limb functional decline |
| Hayashibara 2010 | Mean tender joint count in fall group: 4.30 (SD7.02)  Mean tender joint count in no-fall group: 3.18 (SD 4.88)  p = 0.41 | Unadjusted |
| Ho 1996 | Unadjusted: Bilateral wrist pain:  1.28 (1.13-1.44)  Adjusted: Bilateral lower limb joint pain: 1.4 (1.1-1.8) | age and sex |
| Holt 2011 | 0.80 (0.29-2.19) | Unadjusted |
| Jones 2011 | 5.31 (1.63-17.26) | Unadjusted |
| Kitayuguchi 2015 | One fall:  Model 1: 2.16 (1.02-4.57)  Model 2: 1.50 (0.67-3.39)  For two or more falls:  Model 1: 11.07 (1.43-85.83)  Model 2: 10.79 (1.33-87.19) | Model 1: age, sex, BMI  Model 2: age, sex, BMI, self-rated health, self-reported psychological distress, medication number, gait speed, exercise time |
| Kitayuguchi 2017 | Chronicity and one or more falls:  Both chronic LBP and KP:  Unadjusted: 2.42 (1.29-4.54)  Adjusted: 2.03 (0.95-4.33)  Intensity and one or more falls:  Both chronic LBP and KP:  Unadjusted: 2.99 (1.13-3.53)  Adjusted: 1.57 (0.80-3.05)  Chronicity and two or more falls:  Unadjusted: 1.59 (0.88-2.89)  Adjusted: 2.12 (0.67-6.74)  Intensity and two or more falls:  Unadjusted: 2.55 (1.18-5.53)  Adjusted: 1.58 (0.60-4.17) | age, sex, BMI, community, education years, self-rated health, depression, smoking, chronic disease history, medication use, consultation with physician |
| Leveille 2002 | One or more falls:  Moderate / severe pain lower extremities:  1.27(0.97-1.66)  Widespread pain:  1.66(1.25-2.21)  Recurrent falls:  Moderate /severe pain lower extremities:  1.38(0.93-2.03)  Widespread pain:  1.66(1.10-2.50) | age, race education, BMI, confirmed diseases (hip fracture, angina pectoris, diabetes mellitus, peripheral arterial disease, stroke, Parkinson’s disease), walking disability, previous fall in 12 months before baseline, MMSE score, daily use of psychoactive medications, daily use of analgesic medications, gait speed, balance test score, proxy respondent and follow-up round |
| Leveille 2009 | Multisite pain rate ratios:  Model 1: 1.70(1.34-2.16)  Model 2: 1.71(1.33-2.20)  Model 3: 1.60(1.23-2.06)  Model 4: 1.53(1.17-1.99) | Model 1: socioeconomic characteristics  Model 2: Model 1 plus chronic conditions, physical and cognitive status  Model 3: Model 2 plus physical performance and psychotherapeutic medications  Model 4: Model 3 plus analgesic use and hand and knee arthritis clinical criteria |
| Marshall 2016 | Back pain two sites and any falls (relative risk):  Model 1: RR 1.34  Model 2: RR 1.27(1.12-1.44)  Back pain two sites two or more falls:  Model 1:RR 1.73  Model 2: RR 1.63(1.30-2.05)  Back pain three sites and any falls:  Model 1: RR 1.60  Model 2: RR 1.50(1.23-1.83)  Back pain three sites and two or more falls:  Model 1: RR 1.60  Model 2: RR 1.50(1.23-1.83) | Model 1: age  Model 2 any fall: age, education, smoking, fainting episodes, hip pain, prevalent vertebral fracture  Model 2 two or more falls: age, education, smoking, fainting episodes, hip pain, history of stroke |
| Marshall 2017 | Two pain sites and recurrent falls:  Model 1: RR 1.99  Model 2: RR: 1.50(1.20-1.89)  Two pain sites and any fall:  Model 1: RR: 1.56  Model 2 RR: 1.33(1.16-1.53)  3-5 pain sites and recurrent falls:  Age adjusted RR: 2.78  Multivariable RR: 1.85(1.42-2.42)  3-5 pain sites and any fall:  Age adjusted RR: 1.78  Multivariable RR: 1.40(1.18-1.67) | Model 1: age  Model 2 for recurrent falls: age, dizziness, history of arthritis, knee pain, LUTS, self-related health  Model 2 for any fall: age, dizziness, history of arthritis, knee pain, LUTS, BMI category |
| Oswald 2006 | Per 10 count increase in joint tenderness:  1.2(0.9-1.6) | age |
| Patel 2014 | Prevalence ratio for fall yes/no  1 pain site: 1.21(1.06-1.38)  2 pain sites:1.53(1.31-1.79)  3 pain sites:1.54 (1.30-1.83)  4 pain sites:1.75 (1.51-2.04) | age, sex, ethnicity, education, smoking, BMI, depressive symptoms, obesity, dementia, arthritis, OP, hip fracture, chronic lung disease, myocardial infarction, diabetes mellitus, hypertension, stroke, number of medical conditions, cognitive performance, exercise, frequency analgesic use, chair rise performance, gait speed, grip strength, standing balance performance |
| Stanmore 2013 | Unadjusted: Presence of lower extremity joint tenderness /swelling: 2.0(1.3-2.8)  Model 1: Presence of lower extremity joint tenderness / swelling:  1.7(1.1-2.7)  Model 2: Presence of lower extremity joint tenderness / swelling :  1.7(1.1-2.8) | Model 1: swollen or tender lower extremity joints, DAS28 score, use of psychotrophic medications, taking 4 or more types of medications, taking steroids at baseline, VAS pain score, VAS fatigue score, short FES-I score, HAQ score, Four-Test Balance Scale, Symptoms of feeling dizzy or unsteady, time taken to complete the Chair Stand Test  Model 2: swollen or tender lower extremity joints, DAS28 score, use of psychotrophic medications, taking 4 or more types of medications, taking steroids at baseline, VAS pain score, VAS fatigue score, 12 month history of single fall, 12 month history of multiple falls, history of fracture, history of injuries from previous falls, short FES-I score, HAQ score, Four-Test Balance Scale, symptoms of feeling dizzy or unsteady, time taken to complete the Chair Stand Test |
| Stubbs 2015 | Any fall:  Model1: 3.53(1.97-6.34)  Model 2: 2.36(1.15-4.85)  Model 3: 1.92(0.89-4.13)  Single fall:  Model1: 1.39(0.74-2.59)  Model 2: 0.98(0.44-2.10)  Model 3: 0.78(0.33-1.81)  Recurrent falls:  Model 1: 4.22(2.08-8.56)  Model 2: 3.56(1.46-8.67)  Model 3: 3.43(1.34-8.65) | Model 1: age and sex  Model 2: Model 1 & number of chronic conditions, number of prescribed medications, health related quality of life and timed up and go test  Model 3: Model 2 & physical activity and fear of falling |
