# Supplementary material for: Multisite pain and self-reported falls in older people: systematic review and meta-analysis
Source: Arthritis Res Ther. 2019 Feb 22;21:67. doi: 10.1186/s13075-019-1847-5 (PMC6387492; doi:10.1186/s13075-019-1847-5)
Supplement: Supplementary file 5 — Funnel plot of publications examining the unadjusted relationship between multisite pain and falls for cross-sectional, cohort and case-controlled studies. (docx 22 kb) [file 13075_2019_1847_MOESM5_ESM.docx]

Additional File 5: Funnel plot of publications examining the unadjusted relationship between multisite pain and falls for cross-sectional, cohort and case-controlled studies
